# Effects of adverse childhood experiences and personal resilience on household emergency preparedness: considerations for disaster planning

**DOI:** 10.3389/fpubh.2025.1652564

**Published:** 2025-11-05

**Authors:** Tara Heagele, William Ellery Samuels, Sarah Kaplan, Lisa Wilcox, Taryn Amberson, Charleen McNeill, Lavonne M. Adams

**Affiliations:** ^1^Hunter College of the City University of New York, Hunter-Bellevue School of Nursing, New York, NY, United States; ^2^College of Nursing, East Carolina University, Greenville, NC, United States; ^3^Health Systems and Population Health, University of Washington, Seattle, WA, United States; ^4^College of Nursing and Health Professions, The University of Southern Mississippi, Hattiesburg, MS, United States; ^5^Department of Nursing, Towson University, Towson, MD, United States

**Keywords:** adult survivors of childhood adverse experiences, disaster planning, disasters, preparedness, public health, readiness, resilience psychological

## Abstract

**Introduction:**

Adverse childhood experiences (ACEs) are potentially traumatic events occurring during childhood that can affect adulthood health and wellness, including preparedness for disasters. This study aimed to understand how ACEs, personality traits, personal resilience, and healthcare provider discussions of preparedness affect household emergency preparedness to inform interventions for individuals with a history of ACEs.

**Methods:**

This cross-sectional study was conducted through an internet-based survey of 311 US adults using six evidence-based instruments: ACEs Questionnaire, Big Five Inventory, Chapman University Survey on American Fears, Grit-S, Brief Resilient Coping Scale, and the Household Emergency Preparedness Instrument.

**Results:**

Although most participants had experienced at least one ACE, there was no significant relationship between ACEs and disaster preparedness. Income, confidence in preparedness, and emotional reactivity were more predictive.

**Conclusion:**

Findings can guide the development of tailored community interventions and policies to improve disaster preparedness among individuals with a history of childhood trauma.

## 1 Introduction

The importance of household emergency preparedness is growing as climate change increases the number and severity of disasters triggered and those people affected by them. According to the Center for Research on the Epidemiology of Disasters of the United Nations (1), disaster impacts increased from 1980–1999 to 2000–2019. Reported disasters increased by 3,136, the number of deaths increased by 4 million, and the total number of people affected increased by 780 million. The unpredictable nature of disasters, especially floods, landslides, wildfires, and volcanic activity, necessitate adequate household emergency preparedness to minimize disaster-related morbidity and mortality.

Ferreira et al. (2) found that people (in a US sample) who prepare for disasters are more likely to identify as white, having attained higher education levels, speak English as a first language and exhibit more resilience. In an analysis of households affected by Hurricane Harvey in 2017, Collins et al. (3) found that race, ethnicity, and socioeconomic status were important predictors of the extent to which one was affected by flooding. Cong et al. (4) found that social vulnerability affected preparedness levels, with communities having higher percentages of single-parent households, unemployment, no high school diplomas, and homes with more people than rooms, being less likely to be prepared for disasters. This strongly suggests a mismatch between those who prepare for disasters and those who are at greatest risk.

As environmental calamities continue to escalate in frequency and intensity, already-stressed civil, political, and economic systems are further burdened by additional disaster response and recovery efforts. When considering the adverse effects of disasters on humans and their respective systems, it is challenging to consider the definition of disasters outside of a socio-ecological perspective. Bronfenbrenner (5) supports a bioecological theory of human development, stating “scientifically relevant features of any environment for human development include not only its objective properties but also the way in which these properties are subjectively experienced by the persons living in that environment” (p. 5). Furthermore, scholars identify disasters as high-risk adverse events, capable of disrupting human biophysical and psychosocial systems, as well as socioeconomic systems (6–8).

In light of these chronic stressors that children, families, and communities face, disasters are defined as contextual adverse events with high risk for subjective alteration of an individual’s capacity for biophysical, psychosocial, and financial well-being (9). This definition recognizes disasters as phenomena capable of producing adverse childhood experiences (ACEs), which can lead to childhood alterations in brain development and gene expression (10).

ACEs are defined as potentially traumatic experiences occurring during childhood (aged 0–17 years) that can have lasting effects into adulthood and affect life opportunities. ACEs include exposure to neglect; physical violence or sexual abuse; or having a family member who died by or attempted or suicide (11); parental separation; and living with people with substance use disorders, suffering from depression or mental illness, or incarceration (12). ACEs are associated with adverse physical, psychological, and social outcomes in later life including substance use disorders (13, 14), physical disease (14, 15), family conflict (16), impaired cognitive functioning (13); suicide ideation and attempts (16), and quality of life (14, 17). Approximately 20–48% of children in the United States have experienced at least one ACE (18). While experiencing a disaster as a child has been considered an ACE in recent studies (19, 20), we sought to examine how all ACEs influence household emergency preparedness for disasters as an adult.

Personal resilience has been found to mediate between ACEs and negative outcomes (21, 22). Sassoon et al. (23), for example, found that personal resilience can independently predict health-related quality of life among adults with similar numbers of childhood traumas. Resilience can be expressed as the ability to successfully adapt and adjust to adverse events (24). Resilience has been identified in those who are more likely to prepare for a climate related disaster (2). In qualitative studies conducted by Guzzardo et al. (25) and Heagele (26), some participants who demonstrated household emergency preparedness behaviors attributed these behaviors to their “rough childhoods,” where they “had to learn how to survive young,” suggesting that personal resilience or grit may have a role in preparedness behaviors of adults who have experienced an ACE.

In August 2023, we conducted a review of what is known about ACEs, personal resilience, and household emergency preparedness using the following databases: *MEDLINE Complete*, *CINAHL Complete*, *Health and Psychosocial Instruments*, and *APA PsycArticles*. The terms used in the search included adverse childhood experiences, ACEs, childhood trauma, resilien*, hardiness, disaster preparedness, disaster planning, emergency preparedness, personality development, and personality traits. Exclusion terms included hospital and organization. Eligible materials included English language, peer reviewed articles published between January 2017 and August 2023. The combination of search terms produced 1,498 results, with 43 articles as duplicates. After title and abstract screening, the full text of 12 articles were reviewed. We found no studies examining the relationship between ACEs and disaster preparedness, representing a gap in knowledge.

The aim of this study was to examine the associations between ACEs, personality traits, personal resilience, healthcare provider discussion of disaster preparedness, and household emergency preparedness. We investigated if and how early-life adverse experiences affected later-life inclinations toward household emergency preparedness. We also examined potential associations between healthcare provider discussion of household emergency preparedness and level of preparedness. We thus sought to develop and advocate for household emergency preparedness lessons, community interventions, and public policies to meet the unique needs of community members with a history of childhood trauma.

### 1.1 Research questions and hypotheses (H)

RQ 1: Is there an association between ACEs and level of adulthood household emergency preparedness?

*H*1: Participants who have experienced adversity as children will be able to find and form strategies to help them overcome disaster-related challenges, leading to higher preparedness scores.

RQ 2: Is there an association between healthcare provider discussions of household emergency preparedness and levels of preparedness?

*H*1: Participants who have had a discussion with their healthcare provider about how to prepare for disasters will demonstrate disaster preparedness knowledge, skills, and behaviors, leading to higher preparedness scores.

## 2 Materials and methods

**Figure 1:**
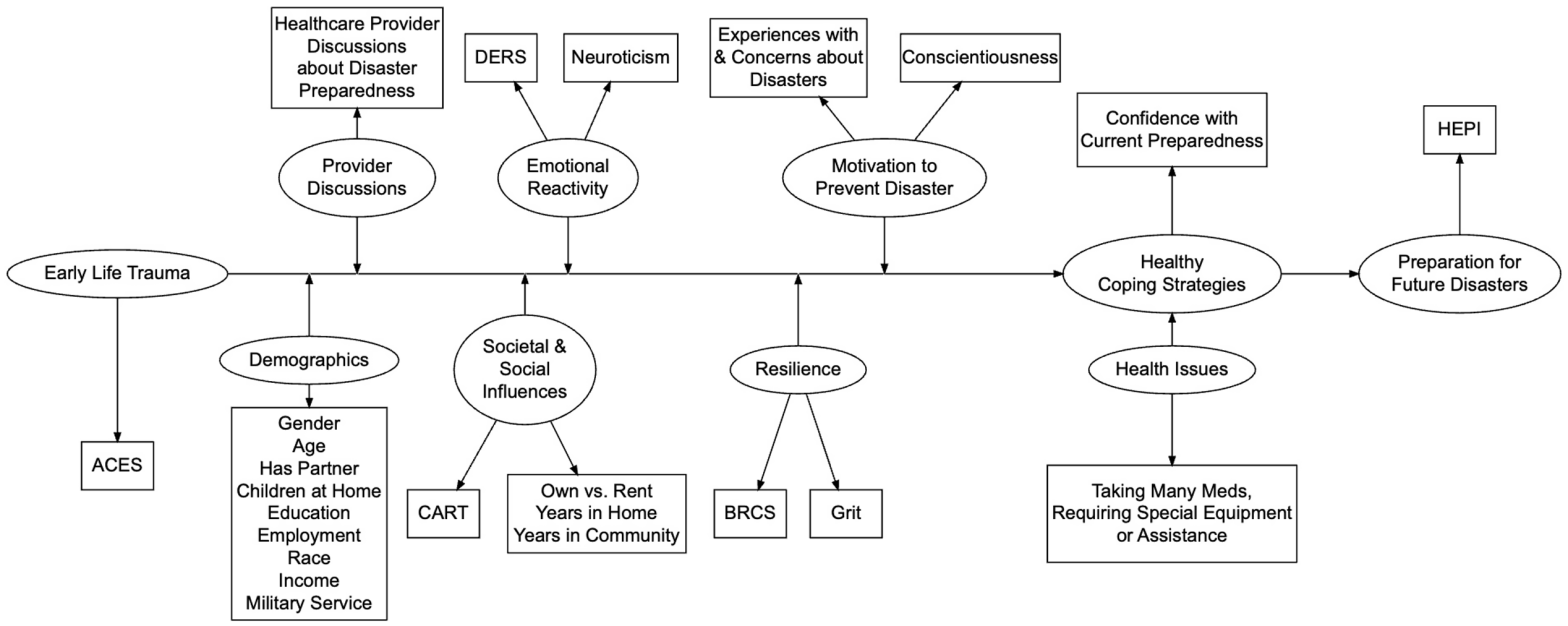
Theoretical model of relationships between the domains and variables used to operationalize them. Hypothesized relationships between adverse childhood experiences, emotional reactivity, motivation, coping, resilience, and household emergency preparedness.

We developed a new model (Figure 1) to use in this study demonstrating how we hypothesized the domains of Debilitation, Participant Demographics, Provider Discussion, Societal and Social Influences, Emotional Reactivity, Resilience, Motivation, Healthy Coping, and Health Issues interrelate to influence household emergency preparedness.

### 2.1 Measurement of the concepts

The ACEs Questionnaire was used to assess the incidence of early life trauma in our participants. The tool was developed by Kaiser Permanente researchers, and research conducted with this tool has found strong, cumulative associations between ACEs and an increased risk of diseases, adult health risk behaviors, and poor health outcomes (27, 28). The current scale contains 17 questions in 10 categories where respondents are scored 1 for ‘yes’ and 0 for ‘no’. Total score can range from 0 to 10.

The Societal and Social Influence domain was constructed using the Connection and Caring domain of the Communities Advancing Resilience Toolkit (CART) (29), home ownership status, years in home, and years in community.

The Big Five Inventory (BFI), a 44-item scale developed by John et al. (30), assesses five personality dimensions of openness, conscientiousness, extroversion, agreeableness, and neuroticism through Likert-scaled responses. The scale has demonstrated acceptable reliability and internal consistencies, ranging between 0.79 and 0.87 (31, 32). In this study, we utilized the questions from the Conscientiousness and Neuroticism dimensions.

The Emotional Reactivity domain was measured using the Neuroticism dimension of the BFI and the Difficulties in Emotional Regulation Scale (DERS-16). The DERS-16 is a 16-item scale adapted by Bjureberg et al. (33) used to measure five aspects associated with difficulties in emotional regulation including nonacceptance of emotional responses, difficulty engaging in goal directed behavior, impulse control difficulties, limited access to emotional regulation strategies, and lack of emotional clarity. Respondents self-rate using five-point Likert-type responses where higher scores indicate higher levels of emotional dysregulation. The DERS-16 demonstrated a high internal consistency in this study (*α* = 0.92).

Although researchers have found that resilience can predict successful reactions to a range of adversities, resilience itself has been differentially defined (34). Given this, our model investigates related concepts that have been found to affect concern and preparation for future challenges, including grit and emotional regulation. Grit is defined as “perseverance and passion for long-term goals” (35), p. 1087. Kannangara et al. (36) found that university students with higher grit scores demonstrated better mental health and self-control. Matthews et al. (37) and O’Neal et al. (38) both report that grit is among the best predictors of success and adaptive responses to acute and chronic stress. We constructed the Resilience domain using the Grit-S and the Brief Resilient Coping Scale (BRCS).

The Grit-S is an 8-item scale adapted from the original 12-item Grit-O developed to measure the concept of grit. The respondent rates the eight statements using a five-point Likert-type scale. Half of the questions address consistency of interest and the other half address perseverance of effort. Testing of the scale has demonstrated acceptable internal consistency. The four samples used to test the adaptation of the Grit-S demonstrated acceptable reliability with internal consistencies ranging between 0.73 and 0.83 (39).

The BRCS is a 4-item scale developed to measure how well respondents adaptively deal with stress. These traits include coping resources, coping with pain, and psychological wellbeing (40). BRCS scores range between 4 and 20 with lower scores denoting less resilience. Kocalevent et al. (41) found that the BRCS has an adequate internal consistency (*α* = 0.76).

The construct of Motivation can be viewed as inspiring one to desire to prevent future recurrences of a past traumatic event. If people with ACEs experience this motivation, it is plausible that carefully prepared and packaged interventions can leverage this construct to inspire these people to become prepared for disasters. Motivation to prepare for disasters was constructed using a subscale of questions from the Chapman University Survey on American Fears (42) and the conscientiousness scale from the BFI. The Chapman subscale was used to measure five aspects associated with preparing for disasters including keeping an emergency supply kit, belief that they will personally experience a disaster in the near future, guilt and confidence relating to disaster preparedness, and belief that a disaster triggered by a natural hazard can cause them serious harm. Respondents rated their agreement with the five statements using a four-point Likert scale ranging from strongly agree to strongly disagree.

The construct of Coping determines how coping strategies one learns from early-life trauma affects how one prepares for disasters. There was no valid and reliable instrument to measure this construct in a disaster context. To measure this mediator, we asked participants one item: I feel that I can do something to keep me safe during disasters (yes/no/unsure).

The Household Emergency Preparedness Instrument (HEPI) General Preparedness scale is an international, all-hazards, comprehensive, 30-item instrument created to measure disaster preparedness of households with support for face, content, and criterion validity of the instrument (43–45). The HEPI questions are objective and ask about what the respondent presently owns or does in a dichotomous format. Higher scores on the HEPI indicate higher levels of household emergency preparedness. The minimum score a respondent could receive on the General Preparedness scale is 0 and the maximum score is 40 (zero points for each no response, one point for each yes response, and two points for each supply item stored in an actual disaster kit). Two subscales comprise the HEPI General Preparedness scale: Preparedness Actions and Planning (PAP) and Disaster Supplies and Resources (DSR). These subscales represent basic emergency preparedness knowledge, behaviors, and actions applicable to all households.

Some of these instruments sample emotionally salient domains that could affect responses to subsequent items. We therefore randomized the order of the instruments for each participant to control for any anchoring or response biases of completing one instrument before any others.

### 2.2 Ethics

The study was approved with exempt status by the Institutional Review Board (IRB) of Hunter College of the City University of New York (protocol #2023-0753-Hunter) on November 29, 2023. Participants provided consent when they enrolled in the study. Participants were not compensated. The data for this study have not been approved to be shared beyond the study team.

### 2.3 Methods

This was an internet-based, social behavioral, quantitative, cross-sectional study of adults residing in the United States including Puerto Rico. Our independent variable was the ACEs Questionnaire score. We assumed that the ACEs Questionnaire measured our model’s Debilitation and Early Life Trauma domain. Covariates, all chosen based on previous household emergency preparedness literature, included military status, functionally diverse (i.e., disability) status, predominant language, age, household composition or familial structure (partner/children), ethnic/national origin identity, place (ZIP code, years in current home, years in community), race, gender identity, education, employment, income, risk perception, prior disaster experience (property damage, injury/illness due to a disaster), dependence on medications, dependence on medical equipment, home ownership, and healthcare provider discussed disaster preparedness. Our hypothesized moderators included Societal and Social Influences, Emotional Reactivity, Resilience, and Motivation to Prepare for Disasters. Our hypothesized mediator was Healthy Coping strategies, as measured by the participants’ household emergency preparedness self-efficacy. Finally, our dependent variable was the HEPI General Preparedness score.

Previous research using the HEPI with a similar population (43) suggested that disaster education interventions can generate large (Cohen’s *d* > 0.8) effects. Assuming *α* = 0.05 and 1–*β* = 0.8, for a linear regression with 15 continuous predictors and one interaction term, we estimated needing a sample size of at least 135 participants.

### 2.4 Recruitment

Inclusion criteria were being aged 18 years or older, understanding and reading English, and living in the US. Participants less than 18 years of age were excluded because children and adolescents are generally not responsible for their household emergency preparedness activities. We only included residents of the US or Puerto Rico because disaster-related policies, resources, and needs vary by country and we shared US-based disaster preparedness educational resources with the participants after they completed the study survey.

Potential participants were recruited via ResearchMatch.org, a National Institutes of Health sponsored “free and secure tool that helps match willing volunteers with eligible researchers and their studies at institutions across the country” (46), para. 1. ResearchMatch emailed their registered volunteers our IRB-approved recruitment script on our behalf. The volunteers had the option either to consent for their contact information to be released to the study team or decline participation. ResearchMatch then released the email addresses of the interested volunteers to the study team. We then emailed the potential participants a link to the survey, starting with an internet-based informed consent form. On December 20, 2023, ResearchMatch emailed our IRB-approved recruitment script to 1,498 of their registered volunteers via random selection. ResearchMatch prevents duplicate potential participants within a four-month period. We randomly selected a new batch of potential participants once a week for 16 weeks, concluding sampling on April 1, 2024, and data collection on April 8, 2024.

### 2.5 Analytic strategy

This study investigated relationships between ACEs, their effects on current dispositions, and the effects of both on actual disaster preparedness. Although an eventual goal in this line of inquiry is to test a structural equation model explicitly examining all of the relationships presented in Figure 1, we did not want to recruit sufficient numbers of participants for that until components of the new model were tested separately.

We conducted such tests through a family of linear regression models predicting total HEPI scores that each added sets of variables related to a respective theoretical domain; we then not only tested whether individual variables significantly predicted disaster preparedness, but also whether that domain significantly contributed to our understanding of preparedness. In addition, there were many moderate correlations between most of the variables; adding related sets of variables provided a theory-based framework for parsing out these relationships to make better sense of them and their impacts.

It is worth noting that the order in which domains are added may affect the extent to which they improve model fits. We chose to add the domains in the order we did for theoretical reasons.

## 3 Results

We randomly selected a total of 23,996 potential participants out of the 135,648 ResearchMatch volunteers registered at the time. We had 754 potential participants consent to be contacted by the study team, with 311 of those participants completing the study, for a response rate of 41.2% of those contacted. This is, however, only 1.3% of the total potential participants.

### 3.1 Descriptive statistics

Participants were adults aged between 23 and 88 years (mean = 49.66, *SD* = 17.07) residing in the US. The number of participants residing in each state is depicted in Appendix A, Figure 2. The majority of participants self-identified as female (*n* = 224, 72.03%), white (*n* = 263, 84.57%), non-Hispanic (*n* = 266, 86.08%), and with education at the baccalaureate (*n =* 114, 36.66%) or graduate (*n* = 119, 38.26%) levels. Most participants reported renting their home (*n =* 201, 64.84%). The average length of time in residence was 10.29 years (*SD* = 10.43, range 23–88) and time in community was 16.16 years (*SD* = 10.16, range 0–68). Approximately 65% (*n* = 203) had a partner and 22% (*n* = 69) had children living in the home. Most (*n* = 265, 85.21%) participants reported that either they or someone in their home took medications daily; 22.51% (*n* = 70) required special equipment; and 13.23% (*n* = 41) required assistance from others. Only 5.47% (*n* = 17) reported that they had discussed disaster preparedness with their healthcare provider. Experiencing illness or injury from a disaster, or knowing someone who did, was positively associated with healthcare provider discussion of disaster preparedness (*r = 0*.21). Complete demographic data of our sample is displayed in Table 1.

**Table 1:** Demographic characteristics of the sample.

Category	Response option	Total responses	*N*	%
Gender	Agender	311	1	0.32
	Cisgender	311	23	7.40
	Fluid	311	1	0.32
	Non-conforming	311	1	0.32
	Queer	311	4	1.29
	Man	311	48	15.43
	Nonbinary	311	4	1.29
	Pangender	311	0	0.00
	Trans	311	2	0.64
	Woman	311	224	72.03
	Something Else	311	3	0.96
Race	African American	311	14	4.50
	American Indian or Alaska Native	311	3	0.96
	Asian	311	9	2.89
	Biracial or Multiracial	311	11	3.54
	Black	311	7	2.25
	Native Hawaiian or Other Pacific Islander	311	0	0.00
	White	311	263	84.57
	Prefer not to Answer	311	4	1.29
Ethnicity	Hispanic	309	11	3.56
	Non-Hispanic	309	266	86.08
	Latinx	309	1	0.32
	Middle Eastern/North African	309	1	0.32
	Unlisted Ethnic Group	309	25	8.09
	Prefer Not to Answer	309	5	1.62
Income	Less than $20,000	295	19	6.44
	$20,000 to $34,999	295	21	7.12
	$35,000 to $49,999	295	39	13.22
	$50,000 to $74,999	295	42	14.24
	$75,000 to $99,999	295	56	18.98
	$100,000 to $149,999	295	63	21.36
	$150,000 or More	295	55	18.64
Education	Less than high school degree	311	1	0.32
	High school degree or equivalent (e.g., GED)	311	8	2.57
	Some college, but no degree	311	50	16.08
	Associate degree	311	19	6.11
	Bachelor’s degree	311	114	36.66
	Graduate degree	311	119	38.26
Employment	Employed, working full-time	311	158	50.80
	Employed, working part-time	311	38	12.22
	Not employed	311	17	5.47
	Retired	311	73	23.47
	Disabled, not able to work	311	25	8.04
Homeowner status	Owns	310	94	30.32
	Rents	310	201	64.84
	Other	310	15	4.84
Household composition	Has partner	311	203	65.27
	Has children in the home	311	69	22.19
Military service	Currently serving or has served in the military	310	21	6.77
Medical needs	Someone in the home has to take medications every day	311	265	85.21
	Someone in the home has a medical condition for which they have to use special equipment	311	70	22.51
	Someone in the home has a disability that requires assistance from others	310	41	13.23
Provider discussion	A healthcare provider has talked to you about disaster preparedness	311	17	5.47
Disaster experience and perceptions	Has personally experienced home damage related to a disaster	310	114	36.77
	Has or knows someone who has experienced an injury or illness due to a disaster	310	59	19.03
	Feels at risk for disasters	311	137	44.05
	Feels that they can do something to keep safe during disasters	311	266	85.53

Although most (*n* = 231, 74.28%) participants reported having experienced at least one ACE, the number of participants experiencing more than one ACE quickly diminished; the mean of the 301 ACEs responses was 2.67 and the median was 2, indicating a positive skew of the distribution, and the relative lack of ACEs among this sample.

HEPI General Preparedness scores (the composite of the PAP and DSR subscales) ranged from 3 to 40 (*n* = 300) with an average of 17.75 (*SD* = 6.59). There was a lower HEPI PAP mean score (*M* = 7.73, *SD* = 4.33) as compared to the HEPI DSR mean score (*M* = 10.02, *SD* = 2.99). Although there were many small to medium correlations between most of the variables, correlations between ACEs and the HEPI score were low to very low and not significant (r_HEPI Composite_ = 0.001, *p* ≈ 1; r_PAP_ = 0.003, *p* ≈ 1; r_DSR_ = −0.014, *p* = 0.814).

Responses from the Chapman University Survey on American Fears scale were used in the constructs of Motivation and Healthy Coping strategies. Full details of the responses are found in Table 2. The response to the item “I feel confident that I know how to prepare for disasters” was positively associated with composite HEPI scores (*r* = 0.49). The question also captured the respondent’s perceptions with ‘I feel that I can do something to keep me safe during disasters’ positively associated with composite HEPI scores (*r = 0.28*).

**Table 2:** Chapman survey of American fears.

Question *N* = 303	Strongly agree	Agree	Disagree	Strongly disagree
By keeping an emergency supply kit, I am improving my chances of surviving a natural or manmade disaster.	129 (42.57%)	155 (51.16%)	18 (5.94%)	1 (0.33%)
I will experience a significant natural or manmade disaster in the near future.	17 (5.61%)	102 (33.66%)	162 (53.47%)	22 (7.26%)
I sometimes feel guilty that I have not done enough to prepare for disasters.	44 (14.52%)	122 (40.26%)	107 (35.31%)	30 (9.90%)
I feel confident that I know how to prepare for disasters.	51 (16.83%)	167 (55.12%)	74 (24.42%)	11 (3.63%)
Natural disasters in my area are capable of doing serious harm to me or my property.	110 (36.30%)	146 (48.18%)	42 (13.86%)	5 (1.65%)

### 3.2 Reliabilities

Inter-item reliabilities (coefficient *α*s) are presented in Table 3. Except for the Chapman University Survey on American Fears Motivation to Prepare for Disaster subscale (α = 0.56), the instruments demonstrated good internal consistency. Note that the Motivation to Prepare for Disaster subscale was not initially created to measure one, unitary dimension, and that we included each item separately here for all theory-based analyses.

**Table 3:** Reliability of instruments used.

Tool	Standardized coefficient ɑ
Chapman Survey of American Fears (Motivation to Prepare for Disaster Subscale)	0.56
Household Emergency Preparedness Instrument – Composite	0.88
HEPI Subscale - Preparedness Action and Planning	0.85
HEPI Subscale - Disaster Supplies and Resources	0.79
GRIT-S	0.84
Brief Resilient Coping Scale	0.73
Difficulty in Emotional Regulation Scale	0.94
Big Five Inventory – Conscientiousness	0.84
Big Five Inventory – Neuroticism	0.86
Communities Advancing Resilience Toolkit: Connection and Caring	0.89

### 3.3 Correlations

Correlations between the major variables are presented in Table 4 (a fuller matrix of correlations is given in Appendix B, Table 7). As those tables show, there were many moderately-sized correlations between most of the major variables. Discounting the correlations between HEPI total and subscores, Neuroticism had the highest average correlations (mean *r* = −0.25) with the other variables followed by DERS (mean *r* = −0.21) and Conscientiousness (mean *r* = −0.11). Correlations with HEPI composite scores ranged from 0.25 (with Conscientiousness) to −0.31 (with Neuroticism), except for CART Connection and Caring scores, with which it did not correlate well (*r* = 0.08). ACEs Questionnaire scores had the lowest average correlation (*x*_Correlations_ = 0.01), correlating best with DERS (*r* = 0.33), Neuroticism (*r* = 0.27), and Grit-S (*r* = −0.25) scores. Correlations with other variables ranged from <0.01 (with HEPI total and PAP) to −0.18 (with CART).

**Table 4:** Major correlations.

Instrument	Subscale	ACEs	HEPI			Grit-S	BRCS	DERS	Big five personality		CART CC
			Total	PAP	DSR				Con.	Neuro.	
HEPI	Total	< 0.01		**0.93**	**0.86**	**0.26**	**0.26**	**−0.29**	**0.25**	**−0.31**	0.08
	Preparedness actions & planning	< 0.01	**0.93**		**0.61**	**0.22**	**0.26**	**−0.25**	0.20	**−0.25**	0.09
	Disaster supplies & resources	−0.01	**0.86**	**0.61**		**0.27**	**0.22**	**−0.30**	**0.28**	**−0.33**	0.06
Grit-S		**−0.25**	**0.26**	**0.22**	**0.27**		**0.29**	**−0.56**	**0.75**	**−0.55**	0.19
Brief resilience scale		−0.10	**0.26**	**0.26**	**0.22**	**0.29**		**−0.34**	**0.25**	**−0.50**	**0.32**
Difficulty in emotional regulation scale		**0.33**	**−0.29**	**−0.25**	**−0.30**	**−0.56**	**−0.34**		**−0.52**	**0.72**	−0.11
Big five personality	Conscientiousness	−0.17	**0.25**	0.20	**0.28**	**0.75**	**0.25**	**−0.52**		**−0.50**	0.18
	Neuroticism	**0.27**	**−0.31**	**−0.25**	**−0.33**	**−0.55**	**−0.50**	**0.72**	**−0.50**		**−0.25**
CART connection and caring subscale		−0.18	0.08	0.09	0.06	0.19	**0.32**	−0.11	0.18	**−0.25**	

Significant correlations (ɑ = 0.05) are presented in bold. CC, Connection and Caring Subscale. Con., Conscientiousness. Neuro., Neuroticism.

### 3.4 Tests of research questions

#### 3.4.1 RQ 1: is there an association between experiencing adverse childhood events and level of household emergency preparedness?

ACEs Questionnaire scores were not significant when used alone in linear regression models predicting HEPI General Preparedness (*β* < 0.01, *SE* = 0.02, *p* = 0.877), PAP (*β* < 0.01, *SE* = 0.02, *p* = 0.841), or DSR scores (*β* < −0.01, *SE* = 0.02, *p* = 0.937). We therefore did not find support for the first research question. Further analyses, described in *Tests of Theoretical Domains* below, did find support for indirect effects of ACES on disaster preparedness, but even there, the effect was relatively weak in this sample.

#### 3.4.2 RQ 2: is there a relationship between healthcare provider discussions of household emergency preparedness and level of preparedness?

Discussions with healthcare providers about household emergency preparedness were also not significantly related to HEPI General Preparedness (*β* = 0.23, *SE* = 0.25, *p* = 0.363), PAP (*β* = 0.35, *SE* = 0.25, *p* = 0.161), or especially DSR scores (*β* < −0.01, *SE* = 0.25, *p* = 0.976). As noted in the descriptive statistics section above, only 17 (5.47%) participants reported having these discussions, suggesting that they are rare, making it difficult to discern their effects here.

### 3.5 Tests of theoretical domains

Table 5 summarizes the changes in how well a model predicted HEPI General Preparedness after the variables of a given domain were added to it. Compared to the base model that only included demographic variables, the model that next included ACEs scores produced a significantly better fit to the data (change in BIC = 8.71, *df* = 1, *p* - 0.003). However, the *term* for ACEs in that second model was itself not significant (*β* = 0.02, *p* = 0.734). Insight into this seeming contradiction can be gained from the correlations between ACEs scores and those demographic variables, which ranged from very small (has a partner, gender-fluid, & non-binary gender *r*s ≈ |0.1|) to medium (income *r* = −0.32); the average correlation between ACEs and demographic variables was 0.11. ACEs thus shares variance with several demographic variables, and with them creates a significantly better prediction of HEPI General Preparedness than demographics alone.

**Table 5:** Changes of model fit for each domain.

Domain	ΔBIC	*p*
Demographics	0	–
ACEs	**8.71**	**0.003**
Provider Discussion	−4.04	~1
Social & Societal	**26.27**	**< 0.001**
Healthy Coping & Health Issues	**50.26**	**< 0.001**
Emotional Reactivity	**19.34**	**< 0.001**
Resilience	**6.66**	**0.040**
Motivation	−4.92	~1

ΔBICs are the changes in Bayesian information criterion when a given domain is added; *p* values indicate whether that domain significantly improved the model fit; the ΔBIC for the Demographic model is 0 since this is set to be the initial (null) model. Bold-faced variables were individually significant in at least one model.

Table 6 presents the parameters for the individual variables for the final model that contained all of the domains; Appendices B, C, Tables 7, 8 present the results for each of the intermediate models, including which variables were significant in each successive model.

**Table 6:** Effect Size (β), standard Error, t, and *p* values for individual variables in the final model.

Domain	Variable	*β*	*SE*	*t*	*p*
Demographics	Gender^1^	< 0.01	0.12	−0.02	0.984
	Age	< 0.01	0.08	−0.02	0.983
	Has Partner	−0.05	0.13	−0.37	0.715
	Children Living at Home	0.04	0.15	0.29	0.771
	Education	−0.08	0.04	−1.82	0.071
	Employed^2^	0.05	0.14	0.38	0.707
	Race^3^	−0.12	0.16	−0.79	0.432
	Non-Hispanic^4^	−0.19	0.16	−1.19	0.234
	Income	**0.09**	**0.04**	**2.41**	**0.017**
	Military^5^	0.18	0.23	0.78	0.438
ACESs	ACES^6^	0.01	0.07	0.14	0.888
Provider discussion	Provider Discussion^7^	0.30	0.27	1.12	0.265
Social & societal	CART^8^	−0.04	0.06	−0.68	0.497
	Owns Home^9^	−0.10	0.13	−0.75	0.455
	Years in Home and Community^10^	0.02	0.06	0.25	0.801
Healthy coping & health issues	Keeping Emergency Kit Helps^11^	**0.15**	**0.06**	**2.56**	**0.011**
	Confident with Disaster Knowledge Preparation^12^	**0.36**	**0.07**	**5.42**	**< 0.001**
	Taking Many Medications^13^	0.01	0.16	0.07	0.944
	Needs Special Equipment^14^	−0.29	0.15	−1.9	0.059
	Requires Assistance^15^	**0.35**	**0.17**	**2.03**	**0.043**
Emotional reactivity	DERS^16^	**−0.16**	**0.09**	**−1.81**	**0.071**
	Neuroticism	−0.02	0.09	−0.19	0.849
Resilience	BRCS^17^	0.03	0.07	0.46	0.649
	Grit	0.08	0.09	0.91	0.364
Motivation	Experienced Damage from a Disaster^18^	0.11	0.13	0.89	0.373
	Experienced Injury/Illness from Disaster^19^	0.12	0.15	0.79	0.430
	Feel at Risk of Disaster^20^	**0.31**	**0.13**	**2.34**	**0.020**
	Expect to Experience a Disaster Soon^21^	−0.01	0.06	−0.11	0.911
	Believe Disasters Can Cause Serious Harm^22^	−0.06	0.07	−0.82	0.414
	Feel Guilty Not Prepared Enough for Disasters^23^	0.11	0.06	1.76	0.079
	Conscientiousness	−0.04	0.08	−0.46	0.645

Bold-faced parameters are significant at α = 0.05. ^1^Female = 1, non-female = 0; ^2^Employed (full- or part-time) = 1, not employed (unemployed, disabled, or retired) = 0; ^3^White = 1, non-white = 0; ^4^Non-Hispanic = 1, Hispanic = 0; ^5^Served or serving in military = 1, did not / is not serving = 0; ^6^Adverse Childhood Events Scale; ^7^Discussed disaster preparations with a healthcare provider = 1; did not discuss = 0; ^8^Communities Advancing Resilience Toolkit Connection and Caring subscale; ^9^Owns home = 1, does not own (rent or “other”) = 0; ^10^Number of years in home plus number of years in community, standardized to z-scores; ^11^Standardized Likert responses to “By keeping an emergency supply kit, I am improving my chances of surviving a natural or man-made disaster”; ^12^Standardized Likert responses to “I feel confident that I know how to prepare for disasters”; ^13^Taking many medications = 1, not taking many meds = 0; ^14^Needs special medical equipment = 1, does not need = 0; ^15^Requires special medical assistance = 1, does not require = 0; ^16^Difficulties in Emotional Regulation Scales; ^17^Brief Resilience Scale; ^18^“Have you personally experienced home damage related to a disaster?,” yes = 1, no = 0; “Have you or someone you know experienced an injury or illness due to a disaster?,” yes = 1, no = 0; ^20^“I feel that I am at risk for disasters,” yes = 1, no = 0;, yes = 1, no = 0; ^21^Standardized Likert responses to “I will experience a significant natural or manmade disaster in the near future”; ^22^Standardized Likert responses to “Natural disasters in my area are capable of doing serious harm to me or my property”; ^23^Standardized Likert responses to “I sometimes feel guilty that I have not done enough to prepare for disasters”.

Many of the relationships that were significant alone became attenuated to non-significance when other variables were added to the models. Among the variables that remained the most reliably predictive of the HEPI General Preparedness score were income, feeling that keeping a kit helps, feeling confident in one’s current preparedness, DERS scores, and feeling at risk for experiencing a disaster. Requiring special medical assistance, military service, age, and education were also often important until many other variables were added. These results may suggest that those who have the means to prepare have indeed done so, or are actively concerned about preparations.

Our participants were often older adults, so it is perhaps not surprising that we did not find a direct relationship among the rather few ACEs they reported experiencing and disaster preparedness; however, ACEs did affect the relationships of demographics on HEPI scores, suggesting that future structural equation models (like those proposed in Figure 1) may prove insightful.

Table 5 also shows the results of adding additional domains of variables beyond ACEs. Neither adding Provider Discussion nor Motivation (e.g., experiences with previous disasters) significantly improved the fit of the overall model. In general, the Motivation domain and provider discussions were found to be distractions, drawing away from our ability to understand disaster preparedness. This may prove to be unique to people from demographics similar to our participants or even just these data, but these results suggest the counter-intuitive conclusion that provider discussions need not be immediately prioritized.

All other domains (Social and Societal, Healthy Coping and Health Issues, Emotional Reactivity, and Resilience) did improve our understanding of General Preparedness. Like the ACEs Questionnaire scores, the resilience domain improved the model fit (χ^2^ = 6.66, *df* = 2, *p* = −0.36) although neither term was significant (β_Grit-S_ = 0.08, *p* = 0.266; β_BRCS_ = 0.06, *p* = 0.387). Both Grit-S and BRCS scores correlated with most of the other major variables (*r*s_Grit-S_ = 0.19–−0.58, *r*s_BRCS_ = 0.22–−0.50), suggesting again that their addition to the model helped primarily through clarifying other relationships (i.e., through partialing out the effects of resilience from other relationships with HEPI scores).

Healthy Coping (as measured by the Chapman University Survey on American Fears items “my kit helps” and “confident I’m prepared”), Health Issues (χ^2^ = 50.26, *df* = 2, *p* < 0.001) and the Societal and Social Influences domains (χ^2^ = 26.27, *df* = 3, *p* < 0.001) were quite important for understanding inclinations toward disaster preparedness. Their contributions to our understanding were significant and large.

Finally, the Emotional Reactivity domain also played an important role (χ^2^ = 19.34, *df* = 2, *p* < 0.001), especially scores on the DERS (β_Final Model_ = −0.17, *p* = 0.035). DERS and Neuroticism scores both significantly correlated with ACEs Questionnaire scores (*r*s = 0.33 & 0.27, respectively), with their combined emotional reactivity domain continuing to make a significant contribution even after ACES scores were added.

## 4 Discussion

We did not find evidence supporting either of our hypotheses. Neither ACEs nor discussions about disasters with one’s healthcare provider significantly predicted disaster preparedness as measured by the HEPI.

Our results revealed rather numerous, but small-to-moderate correlations and limited variability in ACEs, making it difficult to find a subset of factors that reliably predicted preparedness when they were all combined in the initial theoretical model. The respective analyses we could conduct found that most of the theoretical domains (except ACEs and healthcare provider discussions) contributed to our ability to predict preparedness, but that when all components were included together, the welter of moderate interrelationships make it difficult to present a simple, consistent picture, at least among those with few ACEs and relatively secure lives and communities. However, our results do provide some implications for disaster preparedness practice and future research.

### 4.1 Implication for practice: healthcare provider discussion

Our findings differ from studies that found healthcare provider discussions about emergency preparedness can benefit patients’ household emergency preparedness (47, 48). Few participants in our study (*n* = 17, 5.5%), however, indicated discussing household emergency preparedness with their healthcare provider. This finding is consistent with other studies in which few participants and providers indicate discussing household emergency preparedness together (47, 49, 50). The rarity of such communication limits our ability to detect meaningful effects. Although few participants in our study had discussed household emergency preparedness with their healthcare provider, those who did were more likely to have experienced injury or illness from a disaster or to have more health care needs. This is consistent with research indicating that patients identified at high or medium risk for impact from disasters were more likely to receive education on household emergency preparedness than those with low risk or less complex needs (51).

Lack of time is among the reasons why healthcare providers do not consistently initiate household emergency preparedness education with patients (49–51). In our study, those who did have these conversations were more likely to have medical issues that could be affected by disasters, such as requiring assistance with activities of daily living or needing special medical equipment. This suggests that the conversations were at least started to address these needs, whether or not the providers then took the opportunity to expand upon the topic is unknown. It is possible that household emergency preparedness is more “top of mind” for healthcare providers when working with more complex patients; specifically exploring rationale for how healthcare providers prioritize household emergency preparedness education could be valuable in understanding how and when such interactions take place.

We did offer some insight into those whom may be receptive to these sorts of conversations. Responses to the Chapman University Survey on American Fears reflect interest in preventing what participants generally believe are possible and significant disasters, even if they do not believe themselves to be at serious risk. Responses also suggest both concern about not being prepared and belief that preparations can help. Conscientious participants also tended to be better prepared and to generally present more favorable traits, like higher levels of resilience. The Chapman-measured beliefs were associated with the actual levels of preparedness measured by the HEPI. This was especially true of “I sometimes feel guilty that I have not done enough to prepare for disasters” and “I feel confident that I know how to prepare for disasters.”

Adopting an all-people, all-hazards, all-agencies mindset when viewing the ACEs-HEPI theoretical framework for this study, we recognize the key importance of considering subpopulations that may bear a higher burden of risk, such as children experiencing ACEs and adult ACE survivors. Although the majority of participants (74.28%) in this study reported having endured at least one ACE and low levels of household emergency preparedness, this study offers insight on participant characteristics and preparedness behaviors.

### 4.2 Implications for practice: disaster risk perception

Disaster preparedness interventionists, whether they be healthcare providers, public health personnel, community health workers, faith- or community-based leaders, or emergency mangers, can assist community members with understanding their disaster risks by discussing the disasters that are most likely to occur in the community and the causes and prevention of morbidity and mortality outcomes for a recent local disaster. Even if the community members did not experience direct adverse outcomes from the recent local disaster, they can experience them vicariously through these discussions, which then may motivate them to enhance their own preparedness.

The constructs of Motivation and Healthy Coping strategies, as assessed by the Chapman University Survey on American Fears scale, highlight variability in household emergency preparedness behaviors. Despite feeling confident in knowing how to prepare, many participants (40.26%) expressed feelings of guilt or concern about not having done enough to prepare for a disaster. The COVID-19 pandemic exemplified concern and guilt related to the lack of preparedness on many levels, leading to high mortality and morbidity rates, and profound emotional toll across the country (52). Feelings of guilt can be exacerbated if one survives a disaster and their friends and family do not survive (53).

A potentially useful intervention for improving preparedness is motivational interviewing. This is a therapeutic communication technique used to enhance inherent motivation toward specific client goals by “evoking a person’s reasons, desires, and willingness for change using the client’s own speech as a means of clarifying and strengthening their intent” (54), p. 358. Because feeling guilty about not being prepared enough and feeling confident that one knows how to prepare for disasters both predicated higher preparedness levels in this study, interventionists should consider these characteristics when providing disaster preparedness motivational interviewing interventions. Through this technique, the interventionist strives to have the community member come to the conclusions on their own that they may not be fully prepared to endure disaster conditions, that lack of preparedness may impact loved ones or disaster responders if they must be rescued during dangerous storm conditions, and that now, because they have preparedness educational materials from the interventionist, they know how to prepare and can begin preparations. It is plausible that motivational interviewing interventions will result in motivation to start disaster preparedness planning. It is also important to note that motivating community members to equally endorse measurable preparedness planning and disaster supply stockpiling behaviors fosters overall household emergency preparedness. Endorsement of emergency preparedness behaviors contributes to safer post-disaster self-recovery and may minimize the perceived adversity of the disastrous event.

### 4.3 Implications for practice: preparedness self-efficacy

In a recent observational study of US households looking at motivators for disaster preparedness, Miao and Zhang (55) found that recent disaster experience increased participants’ perceived preparedness self-efficacy and propensity to stockpile supplies and make home emergency plans. Conversely, our participants with recent disaster experience were found to be less prepared than the participants who had not experienced a recent disaster. Further research of the effect of recent disaster experience on preparedness self-efficacy is warranted.

Study participants who reported feeling confident in preparing for disasters reported higher levels of preparedness. Similar to the results in this study, Rao et al. (56) reported that individuals who had high confidence in their personal capacity to respond to a disaster had higher overall levels of preparedness. Overconfidence, however, has been viewed as a cognitive bias that impairs an individual’s ability to safely assess when they may experience a future disaster (57). The American Psychological Association (58) describes overconfidence as “an overestimation of one’s actual ability to perform a task successfully, by a belief that one’s performance is better than that of others, or by excessive certainty in the accuracy of one’s beliefs” (para. 1). In this study, over half of the participants (52.1%) disagreed to the following Chapman University Survey on American Fears item, “I will experience a significant natural or manmade disaster in the near future.” Inaccurate disaster predictions, coupled with a lack of household emergency preparedness behaviors, can have devastating individual- and community-level consequences. Interventionists can enhance preparedness self-efficacy by empowering community members with resources and knowledge on how to best prepare for disasters and expressing confidence in the community members’ abilities to make the necessary preparations.

Finally, the current study suggests that those who have the means to prepare have done so or are actively concerned about preparing. These findings are congruent with McNeill et al. (59) where study participants frequently expressed financial resources are a barrier to emergency preparedness. Resolution of this will require equitable emergency preparedness assistance to those without financial resources to purchase emergency preparedness supplies (59) so that existing inequities are not exacerbated after a disaster (60).

### 4.4 Implications for practice: personality traits

In this study, conscientious participants tended to be better prepared and present more favorable traits, like higher levels of resilience. With older or medically frail community members, a focus on client conscientiousness can aid interventionists in assessing how these community members at disproportionate risk for negative disaster impacts might respond to challenges in health, including their likelihood of being prepared for a disaster inclusive of their healthcare needs. The trait of conscientiousness shapes how individuals will respond to such challenges and impacts their behaviors, decisions, and overall well-being, thereby significantly influencing their resilience and health outcomes.

The trait of resilience can aid an understanding of health trajectories in the midst of disasters and emphasize the need for interventionists to focus on mental health of their clients before, during, and after a disaster, particularly the mental health of community members with a history of ACEs. It is critical for interventionists to have a broad view of what constitutes ACEs. Current work in this area focuses not only on ACEs including abuse, violence, incarceration, and homelessness, but also ACEs including poverty, discrimination, violence, poor housing, and lack of opportunity (61). All of these facets of conscientiousness and resilience collectively impact the health and health trajectory of community members.

Emotional reactivity was one of the strongest indicators of concerns about and preparations for disasters. Similar to results found by Reuben et al. (62) and Grusnick et al. (63), we found that ACEs were positively correlated with neuroticism. Emotional reactivity, including neuroticism, was found by Cloitre et al. (64) and Jirakran et al. (65) to mediate the relationship between ACEs and health outcomes of poor physical health, PTSD symptoms, depression, and suicidal behavior. Neuroticism has also been associated with mental health issues, stress, and loneliness (66, 67). We found that higher levels of emotional reactivity were significantly associated with lower levels of household emergency preparedness, identifying another mechanism that excessive emotional reactivity may predispose one to adverse outcomes.

### 4.5 Implications for practice: mental health

With the increasing frequency and intensity of disasters and humanitarian crises, efforts to promote resilience need to be prioritized. Without such efforts, incidence and prevalence of anxiety, depression, post-traumatic stress disorder, complicated grief, and suicidal ideation are expected to increase (68). In fact, the number of people who experience disaster-related mental health issues regularly outweigh those with disaster-related physical injuries (69). In order to mitigate the risks to mental health and resilience at the community level, researchers have proposed the formation of community Resilience Coordinating Networks where “multi-sector coalitions use a public health approach to empower residents to use their existing strengths and resources, and form additional ones, to prevent and heal mental health problems and turn adversities into opportunities to pursue innovative solutions” (69), para. 19. In order to mitigate the risk to mental health and resilience at the individual level, the concept of “healthy hope” can be fostered. Healthy hope is described as the belief that individuals can improve their future by consciously choosing significant personal goals, finding strategies to help them achieve those goals, and mobilizing their willpower to adhere to their plans even when challenges occur (70). Encouraging disaster preparations can foster healthy hope that a disaster can be endured safely and with minimal discomfort.

### 4.6 Strengths and limitations

This was a cross-sectional, observational study with self-reported outcomes and retains the limitations of such a design. We determined *a priori* that an adequate sample size for this study was at least 135 participants. With 311 respondents, we exceeded our desired minimum sample size. Although the sample was of adequate size to find real effects and presents a good geographical representation of the US population, there appears to have been enough self-selection to introduce a bias. Recruitment was conducted from a random selection of a national sample, but potential participants self-enrolled. It is likely that these participants tended to be those who are already more concerned about disaster preparedness. Our sample was more affluent, educated, and urban than the general population, and renters were overrepresented. Affluent households may have additional resources available to undertake preparedness actions. Apartment dwellers may encounter significant barriers to stockpiling supplies because of restricted storage capacity. Additionally, as tenants, renters may have little control over building-level preparedness managed by property owners or staff. Gender and race perspectives may also have been limited in our findings since white women were overrepresented.

Self-report and social-desirability bias are also potential threats to validity. However, to minimize these threats, respondents self-reported anonymously to an internet survey, not a live person, and we assured them that there were no right or wrong answers, potentially decreasing social desirability bias. Possible information bias may have occurred from respondent burden due to the length of the survey. To overcome this bias, we added an “are you still paying attention” question in the survey, which was correctly answered by all of the participants.

A final limitation to consider involves potential recall bias because the participants answered questions about their past experiences with disasters and ACEs.

### 4.7 Implications for research

Our research questions could be examined with a stronger study design, using randomness, confirmation of self-report variables, and prospective data collection. Researchers can also consider using the Social-Ecological Model of Risk and Resilience in future research (71). The socioecological systems in which people exist could be relevant to looking at disaster preparedness, resilience, and ACEs, given the relationship of income and social capital to preparedness levels, and that higher prevalence rates of ACEs are noted among populations with less education or income levels and in socially marginalized groups (72).

The survey did not distinguish between who initiated the healthcare provider/patient discussion of household emergency preparedness, so we do not know if participants perceived themselves to be at greater risk and thus sought out additional information, or if the healthcare provider perceived the participant to be at greater risk and thus prioritized this conversation. Although research has not consistently shown a significant positive relationship between healthcare provider discussion and promotion of household emergency preparedness and their patients’ household emergency preparedness, the rarity of such interactions may affect the ability to detect significance. Additional research into mitigation of barriers to meaningful dialog and educational strategies is warranted so that the potential to enhance health through household emergency preparedness is not overlooked.

Exposure to disaster events should continue to be evaluated as an ACE to determine associations with physical and emotional health outcomes in adulthood. Additional model inquiry with a larger sample can test a structural equation model explicitly testing all of the relationships, strengthening future use of the model.

## 5 Conclusion

The results of our study suggested that an indirect sense of disaster preparedness self-efficacy predicted preparedness. This indicates that a disaster preparedness interventionist could motivate people to prepare for disasters by instilling effort optimism, meaning the interventionist helps the participant develop strong beliefs that the effort of developing an evacuation and emergency communication plan and assembling a disaster supply kit will pay off by minimizing the discomforts of disaster conditions.

Healthcare providers, across in- and out-patient settings, should engage in disaster preparedness conversations with all patients as part as primary prevention for disaster-related morbidity and mortality and secondary prevention for re-traumatization. There are many free, online disaster preparedness resources from reputable organizations that could be downloaded and printed/posted in exam and waiting rooms, provided with discharge instructions, and/or sent via web-based patient portals.

Our participants with characteristics that may increase their risk for adverse disaster-related impacts (had experienced a recent disaster, renter, unemployed, or retired) were found to be less prepared. It would be well worth increasing disaster preparedness educational efforts and community resources in disadvantaged or marginalized communities. Enhancing local community-level emergency preparedness efforts by building individual-level disaster risk reduction capacities decreases the risk of re-traumatizing populations that may bear a higher burden of risk for negative disaster-related impacts, such as children experiencing ACEs and adult ACE survivors. Additionally, working with policy makers to advocate for equitable assistance to those without adequate financial resources to properly prepare for emergencies would likely be of great benefit for those in greatest need. Healthcare and academic institutions are key community-based agencies that can promote local-level emergency preparedness educational programs to improve residents’ confidence and resilience in preparing for disasters, with a goal of minimizing post-disaster related stress.

The value of household emergency preparedness will increase as a greater number of disasters occur due to climate change. Identifying factors that promote or prevent disaster preparedness across a variety of populations is essential to improve preparedness efforts. Although our results did not demonstrate the expected associations between ACEs, provider discussion, and disaster preparedness, this study proposes a framework for examining disaster preparedness behaviors and potential moderators in those with a history of ACEs.

## Data Availability

The datasets presented in this article are not readily available because the participants did not consent for their data to be shared beyond the study team. Requests to access the datasets should be directed to Tara Heagele, th1591@hunter.cuny.edu.
