# Supplementary material for: Effects of adverse childhood experiences and personal resilience on household emergency preparedness: considerations for disaster planning
Source: Front Public Health. 2025 Nov 5;13:1652564. doi: 10.3389/fpubh.2025.1652564 (PMC12627066; doi:10.3389/fpubh.2025.1652564)
Supplement: Supplementary file 1 [file Data_Sheet_1.PDF]

Effects of Adverse Childhood Experiences and Personal Resilience on Household Emergency Preparedness: Considerations for Disaster Planning

Supplementary Figure and Tables

Appendix A

Figure 2

Participation by State

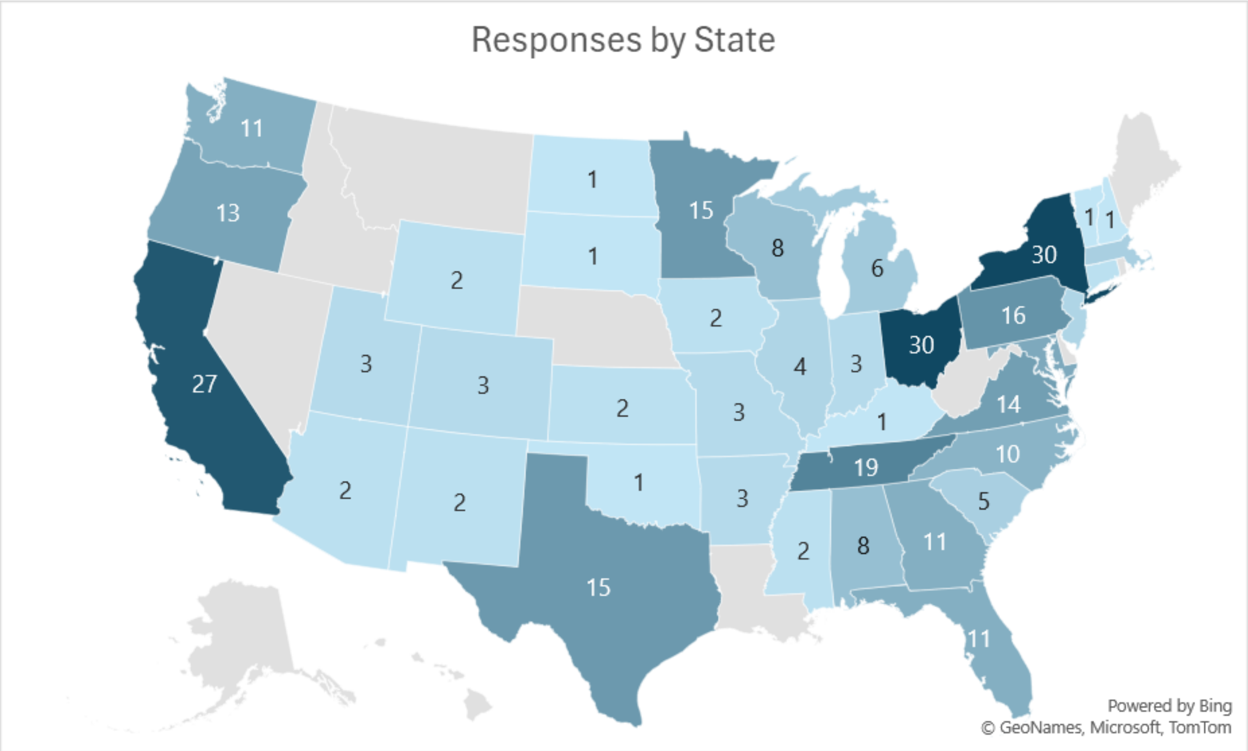

Note: Participants were 311 adults residing in the US. The darker the color, the higher concentration of participants.

Appendix B

Table 7

Tests of Changes in Model Fits and of Individual Variables

	**Demographics Alone**		**Adding ACEs**		**Adding Provider Discussion**		**Adding Social & Societal**		**Adding Healthy Coping & Health Issues**		**Adding Emotional Reactivity**		**Adding Resilience**		**Adding Motivation**	
**Change in Model Fit**				***p***		***p***		***p***		***p***		***p***		***p***		***p***
			**8.71**	**.003**	-4.04	~1	**26.27**	**< .001**	**50.260**	**< .001**	**19.34**	**< .001**	**6.66**	**0.04**	-4.92	~1
**Tests of Individual Variables**																
**Variable**	**β**	***p***	**β**	***p***	**β**	***p***	**β**	***p***	**β**	***p***	**β**	***p***	**β**	***p***	**β**	***p***
Gender[1]	.06	.625	.06	.652	.06	.635	.06	.660	-.05	.674	-.05	.691	-.05	.638	< .01	.984
Age	**.16**	**.033**	**.15**	**.040**	**.15**	**.042**	.12	.127	.06	.414	-.01	.885	-.02	.766	< .01	.983
Has Partner	.21	.126	.18	.197	.17	.215	.14	.346	.03	.792	-.02	.846	-.04	.745	-.05	.715
Children Living at Home	-.08	.593	-.09	.585	-.12	.434	-.10	.551	-.08	.586	-.05	.744	.01	.956	.04	.771
Education	-.08	.056	-.08	.054	**-.09**	**.042**	-.09	.053	-.06	.125	-.05	.241	-.06	.137	-.08	.071
Employed[2]	.06	.705	.06	.693	.07	.641	.06	.674	.06	.657	.03	.806	.03	.819	.05	.707
Race[3]	-.07	.672	-.06	.728	-.06	.733	-.03	.870	-.07	.655	-.08	.588	-.10	.513	-.12	.432
Non-Hispanic[4]	-.18	.287	-.19	.263	-.20	.241	-.17	.350	-.19	.241	-.20	.211	-.14	.400	-.19	.234
Income	**.08**	**.050**	**.09**	**.037**	**.09**	**.030**	**.09**	**.032**	**.11**	**.006**	**.09**	**.014**	**.10**	**.010**	**.09**	**.017**
Military[5]	**.58**	**.018**	**.57**	**.024**	**.58**	**.021**	.45	.083	.25	.276	.22	.333	.22	.350	.18	.438
ACES[6]			.02	.734	.02	.756	.03	.703	.01	.899	.08	.187	.07	.310	.01	.888
Provider Discussion[7]					.32	.221	.39	.177	.12	.632	.25	.326	.30	.258	.30	.265

CART[8]							.09	.183	.03	.613	-.02	.761	-.03	.567	-.04	.497
Owns Home[9]							-.13	.392	-.09	.485	-.12	.380	-.10	.463	-.10	.455
Years in Home and Community[10]							.04	.611	.03	.680	.04	.477	.03	.629	.02	.801
Keeping Emergency Kit Helps[11]									**.16**	**.005**	**.16**	**.005**	**.14**	**.012**	**.15**	**.011**
Confident with Disaster Knowledge Preparation[12]									**.43**	**< .001**	**.40**	**< .001**	**.41**	**< .001**	**.36**	**< .001**
Taking Many Medications[13]									.02	.883	.09	.541	.10	.531	.01	.944
Needs Special Equipment[14]									-.17	.242	-.14	.308	-.16	.282	-.29	.059
Requires Assistance[15]									.21	.214	.27	.107	.30	.079	**.35**	**.043**
DERS[16]											**-.16**	**.040**	-.13	.133	-.16	.071
Neuroticism											-.03	.678	< .01	.979	-.02	.849
BRCS[17]													.06	.387	.03	.649
Grit													.08	.266	.08	.364
Experienced Damage from a Disaster[18]															.11	.373
Experienced Injury/Illness from Disaster[19]															.12	.430
Feel at Risk of Disaster[20]															**.31**	**.020**
Expect to Experience a Disaster Soon[21]															-.01	.911

Appendix C

Table 8
Summary of Model Tests

**Model/ Added Domain**	**1**	**2**	**3**	**4**	**5**	**6**	**7**	**8**
Demo-graphics	· Age<br>· Income<br>· Military	· Age<br>· Income<br>· Military	· Age<br>· Education<br>· Income<br>· Military	· Income	· Income	· Income	· Income	· Income
**ACEs**								
Provider Discussion								
**Social & Societal**								
**Healthy Coping & Health Issues**					· Keeping Emergency Kit Helps<br>· Confident with Disaster Knowledge Preparation	· Keeping Emergency Kit Helps<br>· Confident with Disaster Knowledge Preparation	· Keeping Emergency Kit Helps<br>· Confident with Disaster Knowledge Preparation	· Keeping Emergency Kit Helps<br>· Confident with Disaster Knowledge Preparation<br>· Requires Assistance
**Emotional Reactivity**						· DERS		
**Resilience**								
Motivation								· Feel at Risk of Disaster

Note: Bold-faced domains significantly improved the model fit; listed variables were significant in a given model; empty cells indicate that no individual variables were significant, regardless of improvements in model fits.
